# Fetal Loss and Preterm Birth Caused by Intraamniotic *Haemophilus influenzae* Infection, New Zealand

**DOI:** 10.3201/eid2809.220313

**Published:** 2022-09

**Authors:** Thomas Hills, Caitlin Sharpe, Thomas Wong, Tim Cutfield, Arier Lee, Stephen McBride, Matthew Rogers, May Ching Soh, Amanda Taylor, Susan Taylor, Mark Thomas

**Affiliations:** Medical Research Institute of New Zealand, Wellington, New Zealand (T. Hills);; Auckland District Health Board, Auckland, New Zealand (T. Hills, C. Sharpe, A. Taylor, M. Thomas);; Waitematā District Health Board, Auckland (T. Hills, T. Cutfield, M. Rogers);; Counties Manukau District Health Board, Auckland (C. Sharpe, T. Wong, S. McBride, M.C. Soh, S. Taylor);; University of Auckland, Auckland (A. Lee, M. Thomas)

**Keywords:** Haemophilus influenzae, bacteria, intraamniotic, infection, fetal loss, preterm birth, pregnancy, placenta, bacteremia, sepsis, maternal sepsis, neonatal sepsis, sepsis in pregnancy, New Zealand

## Abstract

Invasive *Haemophilus influenzae* infection during pregnancy can cause preterm birth and fetal loss, but the mechanism is unclear. We investigated 54 cases of pregnancy-associated invasive *H. influenzae* disease in 52 unique pregnancies in the Auckland region of New Zealand during October 1, 2008‒September 30, 2018. Intraamniotic infection was identified in 36 (66.7%) of 54 cases. Outcome data were available for 48 pregnancies. Adverse pregnancy outcomes, defined as fetal loss, preterm birth, or the birth of an infant requiring intensive/special care unit admission, occurred in 45 (93.8%) of 48 (pregnancies. Fetal loss occurred in 17 (35.4%) of 48 pregnancies, before 24 weeks’ gestation in 13 cases, and at >24 weeks’ gestation in 4 cases. The overall incidence of pregnancy-associated invasive *H. influenzae* disease was 19.9 cases/100,000 births, which exceeded the reported incidence of pregnancy-associated listeriosis in New Zealand. We also observed higher rates in younger women and women of Māori ethnicity.

## Abstract 2

*H. influenzae* is as a rare but major cause of pregnancy-associated invasive disease.

*Haemophilus influenzae* serotype B (Hib) causes a range of clinical syndromes, including pneumonia, primary bacteremia, and meningitis (*1*,*2*). Childhood immunization with conjugated Hib vaccines has resulted in dramatic decreases in illness and death attributable to Hib (*2**–**4*). Most invasive *H. influenzae* disease is now caused by nontypeable *H. influenzae* (NTHi) which predominantly affects young children and the elderly (*2*,*5*,*6*). In industrialized countries, deaths caused by NTHi infection are now more common than deaths caused by Hib infection (*6*).

Pregnancy is associated with a 17-fold increase in the incidence of invasive *H. influenzae* infection, largely caused by infection with NTHi (*7*). Invasive *H. influenzae* infection during the first 24 weeks of pregnancy is associated with >90% rate of fetal loss (*7*). Beyond 24 weeks gestation, premature birth occurred in 8 (28.6%) of 28 case-patients and stillbirth in 2 (7.1%) of 28 case-patients (*7*). The burden of NTHi infection extends into the neonatal period, resulting in a high incidence of invasive disease in the first 28 days of life, especially in extremely premature neonate; incidence of invasive NTHi infection is 365-fold higher for neonates at <28 weeks’ gestation than for term neonates (>36 weeks’ gestation) (*5*,*8*,*9*).

Literature describing the burden of pregnancy-associated invasive *H. influenzae* infection consists largely of case reports and public health surveillance data (*7**,**9**‒**11*). Studies have been limited by a paucity of genital tract or postmortem microbiologic data. The mechanisms of preterm birth and fetal loss associated with invasive *H. influenzae* infection are incompletely understood. Historically, *H. influenzae* has not been recognized as a leading cause of intraamniotic infection (IAI) (*12*). However, recent case reports describe IAI that showed histologic evidence of acute necrotizing chorioamnionitis, suggesting that maternal *H. influenzae* infection can involve the amniotic cavity and the fetus (*13*).

We report 10 years of pregnancy-associated invasive *H. influenzae* infection in Auckland, New Zealand. We focus on the overall disease burden and the mechanisms of adverse pregnancy outcomes.

## Methods

We identified cases of invasive *H. influenzae* disease during a 10-year period (October 1, 2008‒September 30th, 2018) from the hospital laboratory records of Auckland City Hospital, North Shore Hospital, Waitakere Hospital, and Middlemore Hospital, which provide free healthcare to the population of the Auckland region (resident population ≈1.7 million persons in 2018). We searched the computerized records of the 3 microbiology laboratories serving these hospitals to identify all patients who fulfilled the US Centers for Disease Control and Prevention criteria for *H. influenzae* invasive disease: isolation of *H. influenzae* from >1 samples collected from a normally sterile site (e.g., blood, cerebrospinal fluid, placental tissue) (*14*). In New Zealand, maternity care is delivered through a network of primary, secondary, and tertiary birthing facilities that, in the Auckland region, are served by these 3 microbiology laboratories. Home births are uncommon, accounting for 3.4% of births (*15*). All neonatal hospital-level care, such as care that would be required for neonates who have *H. influenzae* disease, is delivered in the study hospitals.

We reviewed electronic health records for all cases of invasive disease to identify maternal invasive *H. influenzae* infections, defined as case-patients from whom *H. influenzae* was isolated from samples from pregnant women, and neonatal invasive *H. influenzae* infections, defined as case-patients from whom *H. influenzae* was isolated from samples from infants in the first 28 days of life. Taken together, these case-patients constituted the pregnancy-associated invasive *H. influenzae* study population. Neonatal cases were considered early onset if *H. influenzae* was identified from samples taken within 48 hours of birth. 

We extracted prioritized ethnicity, area-level New Zealand deprivation index (NZDep2013; index of socioeconomic deprivation based on maternal location of residence at the time of delivery) (*16*), maternal age, gestation, microbiologic and prespecified clinical outcome data (pregnancy outcome, death at 30 days, and death at 12 months) from the electronic health records. We grouped NZDep2013 index data into quintiles (1 = least socioeconomic deprivation area, 5 = most socioeconomically deprived area). We defined term birth as delivery at >37 weeks’ gestation. Whether *H. influenzae* isolates were Hib was determined by testing performed at the Invasive Pathogens Laboratory at the Institute of Environmental Science and Research (Porirua, New Zealand).

Antimicrobial susceptibility testing of all isolates was performed in the hospital laboratories by using accredited methods from the European Committee on Antimicrobial Susceptibility Testing (https://www.eucast.org) or Clinical Laboratory Standards Institute (https://www.clsi.org). We extracted data on susceptibility test results from the laboratory records for each isolate.

We categorized cases as intraamniotic infection when *H. influenzae* was cultured from placental tissue, products of conception, or high vaginal swab specimens for case-patients who had concurrent *H. influenzae* bacteremia. We categorized cases as pneumonia if the clinical diagnosis was pneumonia or if chest radiography during the same hospital admission was reported as demonstrating pneumonia. We categorized cases were as meningitis if the clinical diagnosis was meningitis or if *H. influenzae* was isolated from cerebrospinal fluid. We categorized case-patients who had >1 positive blood culture as having primary bacteremia when the documented clinical impression did not specify an alternative clinical syndrome (such as meningitis or pneumonia) and cases could not be otherwise categorized by other microbiologic culture results.

Birth rate and demographic data for the Auckland region during the study period were provided by Statistics New Zealand as a customized data extract to enable calculation of incidence rates. We used relative risk from univariate and multivariate Poisson regression with ethnicity, age, and NZDep2013 in a regression model to look for an association between ethnicity, age, or deprivation and pregnancy-associated invasive *H. influenzae* disease. We performed statistical analyses by using SAS version 9.4 (SAS Institute Inc., https://www.sas.com). The study was approved by the Auckland Health Research Ethics Committee (AHREC 000103).

## Results

We identified 54 cases of pregnancy-associated invasive *H. influenzae* disease: 38 (70.4%) maternal cases and 16 (29.6%) neonatal cases. In 2 pregnancies, the mother and the neonate both had invasive *H. influenzae* disease; therefore, the 54 index cases resulted from 52 unique pregnancies.

### Case Demographics

Of the 52 women who had maternal *H. influenzae* disease, who gave birth to an infant with neonatal disease, or both, most (77%) were of Māori or Pacific descent (Table 1). All 16 neonatal cases were early-onset *H. influenzae* infection. Socioeconomic deprivation data were available for 48 cases; 26 (54.2%) cases were in women living in areas with the most deprived NZDep2013 quintile score. The median gestation for the neonatal case-patients was 34 weeks (range 26–41 weeks), and the median age of the mother at the time of delivery was 29.5 years (range 18–43 years) (Table 1). Maternal age was unavailable for 2 neonatal case-patients. The median gestation for maternal case-patients was 32 weeks (range 8–40 weeks), and the median age of the women at the time of diagnosis was 25 years (range 15–44 years).

**Table 1:** Pregnancy-associated invasive *Haemophilus influenzae* case demographic and clinical data, New Zealand*

Variable	Maternal cases	Neonatal cases	Total pregnancies
Total	38	16	52†
Maternal ethnicity			
European	4 (10.5)	5 (31.3)	8 (15.4)
Māori	17 (44.7)	4 (25)	20 (38.5)
Pacific	14 (36.8)	6 (37.5)	20 (38.5)
Other	3 (7.9)	1 (6.3)	4 (7.7)
Median maternal age, y (range)‡	25 (15–44)	29.5 (18–43)	25 (15–44)
Socioeconomic deprivation‡			
Quintile 5	22 (59.5)	6 (46.1)	26 (54.2)
Quintile 4	10 (27.0)	2 (15.4)	12 (25.0)
Quintile <3	5 (13.5)	5 (38.5)	10 (20.8)
Median gestation,‡ wk (range)	32 (8–40)	34 (26–41)	32 (8–41)
Pregnancy outcomes			
Intrauterine death <24 weeks’ gestation	13 (34.2)	0	13 (25.0)
Intrauterine death >24 weeks’ gestation	3 (7.9)	1 (6.3)	4 (7.7)
Live preterm birth	13 (34.2)	10 (62.5)	21 (40.4)
Live birth at term	6 (15.8)	4 (25)	10 (19.2)
Pregnancy outcome unclear	3 (7.9)	1 (6.3)	4 (7.7)
Clinical diagnosis			
Intraamniotic infection§	34 (89.5)	2 (12.5)	36 (69.2)
Primary bacteremia	4 (10.5)	12 (75.0)	14 (26.9)
Pneumonia	0	1 (6.3)	1 (1.9)
Meningitis	0	1 (6.3)	1 (1.9)
Other	0	0	0
Specimens culturing *H. influenzae*¶			
Maternal blood culture	13	1	14
Neonatal blood culture	2	15	17
Placental tissue or products of conception	30	3	33
High vaginal swab specimens§	17	0	17
Cerebrospinal fluid	0	1	1

*Values are no. (%) unless otherwise indicated. †In 2 pregnancies, invasive *H. influenzae* infection occurred in the mother and the neonate. Thus, the 38 maternal cases and 16 neonatal cases occurred in 52 unique pregnancies. ‡Data for gestational duration were unavailable for 2 neonatal cases and 1 maternal case; maternal age data were unavailable for 2 cases; 2013 area-level New Zealand deprivation index (NZDep2013) data were unavailable for 2 cases. NZDep2013 quintile 5 is the most deprived socioeconomic area, and quintile 1 is the least deprived socioeconomic area. §Intraamniotic infection was defined as *H. influenzae* isolated from placental samples or products of conception (with or without maternal/neonatal *H. influenzae* bacteremia) or maternal *H. influenzae* bacteremia with *H. influenzae* concurrently isolated from high vaginal swab specimens. Isolation of *H. influenzae* from a high vaginal swab specimen alone, without isolation from another site, was insufficient to meet the case definition for invasive *H. influenzae* disease. Invasive disease was diagnosed in 9 cases with associated maternal bacteremia, 1 case with associated neonatal bacteremia, and 7 cases with documented infection of placental tissue. ¶The total number of positive culture results exceeds the number of cases because in some cases *H. influenzae* was isolated from >1 site.

### Sites of Infection

**Figure 1:**
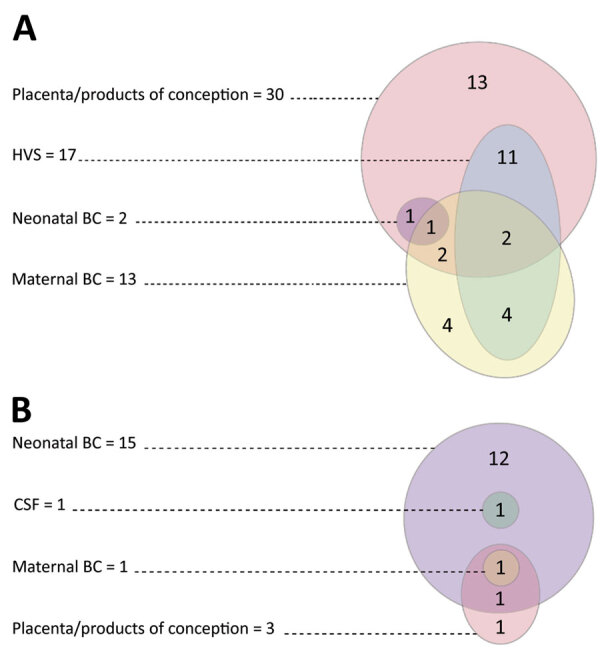
Sites from which *Haemophilus influenzae* was isolated in maternal cases (A) and neonatal cases (B), New Zealand. Overlapping colored circles and ovals indicate multiple types of samples collected from the same cases. BC, blood culture; CSF, cerebrospinal fluid; HVS, high vaginal swab.

We identified IAI in 36 (66.7%) of 54 cases of pregnancy-associated invasive *H. influenzae* infection: 34 (89.5%) of 38 maternal cases and 2 (12.5%) of 16 neonatal cases (Table 1). *H. influenzae* was isolated from placental tissue or products of conception in 33 IAI cases and from maternal or neonatal blood cultures with concurrent isolation from cervical or high vaginal swab specimens in 3 IAI cases (Figure 1).

### Microbiologic Characteristics

Typing data were available for 26 isolates, predominantly from cases with bacteremia. Isolates other than those from blood cultures were not routinely sent for typing. Hib was identified in only 1 case. Isolates were not serotypeable for 18/26 cases and confirmed not to be Hib by molecular testing in 7/26 cases (but not further typed by molecular or serologic methods). The proportion of isolates found to be antimicrobial susceptible was 45/53 (84.9% of tested isolates) for amoxicillin, 54/54 (100% of tested isolates) for amoxicillin/clavulanate, 50/52 (96.2% of tested isolates) for cefuroxime, and 33/47 (70.2% of tested isolates) for sulfamethoxazole/trimethoprim.

### Pregnancy Outcomes

Pregnancy outcome data were available for 48/52 (92.3%) pregnancies. Fetal loss occurred in 17/48 (35.4%) pregnancies, before 24 weeks’ gestation in 13 cases and after 24 weeks’ gestation in 4 cases. An additional 21/48 (43.8%) pregnancies resulted in preterm birth. Of those, 20 (95.2%) required admission to a neonatal intensive care unit (NICU) or special care baby unit (SCBU) and 1 died (described earlier). The remaining 10 pregnancies resulted in birth at term, but 7 of those neonates required admission to an NICU or SCBU. Therefore, adverse pregnancy outcomes, defined as fetal loss, preterm birth, or birth of an infant requiring NICU/SCBU care, occurred in 45/48 (93.8%) of the pregnancies for which outcome data were available. Only 3/48 (6.3%) affected pregnancies resulted in a live birth of an infant not requiring NICU/SCBU care.

### Mortality Rate Outcomes

One (6.25%) of 16 neonatal case-patients, an infant born at 26 weeks’ gestation, had *H. influenzae* bacteremia diagnosed in the first 24 hours of life and died shortly thereafter. None of the 38 maternal case-patients died, although 30-day and 1-year outcome data were unavailable for 1 maternal case-patient (Table 1). Similarly, no mothers of neonatal case-patients died, although 30-day and 1-year outcome data were unavailable for the mother of 1 neonatal case-patient.

### Epidemiology

**Figure 2:**
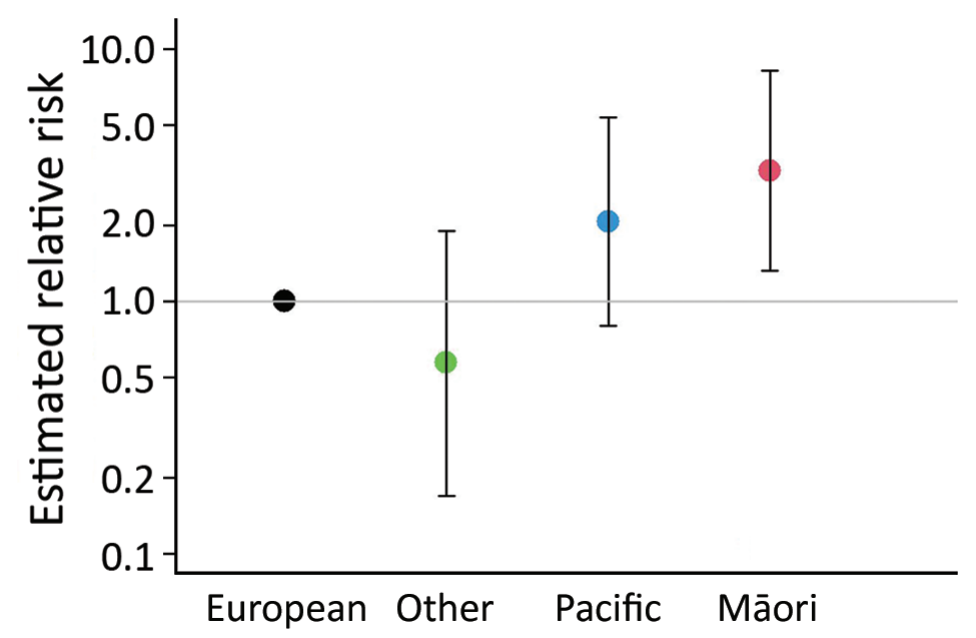
Relative risk for pregnancy-associated invasive *Haemophilus influenzae* infection, by ethnicity, in a multivariable regression model, New Zealand. Error bars indicate 95% CIs.

During the study period, there were 241,653 births in the Auckland region. Complete demographic data were available for 48/52 pregnancies, so we used those 48 pregnancies to calculate an overall incidence pregnancy-associated invasive *H. influenzae* disease rate of 19.9 cases/100,000 births. The rate varied greatly by maternal ethnicity; 53.7 cases/100,000 births for Māori women, 33.6 cases/100,000 births for Pacific women, 9.0 cases/100,000 births for women from Europe, and 5.86 cases/100,000 births for women of other ethnicities (Table 2). Incidence was highest in the youngest maternal age group (<19 years), 65.1 cases/100,000 births, and decreased progressively to 8.8 cases/100,000 births in the oldest maternal age group (>35 years). Ethnicity, age group, and sociodemographic deprivation were each significantly associated with the incidence of pregnancy-associated invasive *H. influenzae* disease by univariable analyses (p<0.0001 for each). In a multivariable regression model (Table 2), ethnicity was significantly associated with the risk for pregnancy-associated invasive *H. influenzae* disease (p = 0.0035), whereas age group (p = 0.1115) and socioeconomic deprivation (0.1015) were not associated. Compared with women from European, the relative risk of infection for Māori women was 3.28 (95% CI 1.32–8.19) and the relative risk for Pacific women was 2.07 (95% CI 0.80–5.37) (Figure 2).

**Table 2:** Pregnancy-associated invasive *Haemophilus influenzae* incidence by maternal ethnicity and age and socioeconomic status, New Zealand*

Variable	Births, no. (%)	Cases, no. (%)	Crude incidence per 100,000 births (95% CI)	Poisson regression relative risk (95% CI)
Maternal ethnicity				
Māori	37,218 (15.4)	20 (42)	53.74 (33.50–80.84)	3.28 (1.32–8.19)
Pacific	47,655 (19.72)	16 (33)	37.58 (19.70–52.81)	2.07 (0.80–5.37)
Other	68,268 (28.25)	4 (8)	5.86 (1.82–13.61)	0.57 (0.17–1.90)
European	88,512 (36.63)	8 (17)	9.04 (4.13–16.82)	Referent
Total	241,653 (100.00)	48 (100.00)	19.86	NA
Maternal age, y				
<19	10,758 (4.45)	7 (15)	65.07 (27.96–125.80)	3.19 (0.97–10.48)
20–24	36,498 (15.1)	17 (35)	46.58 (27.80–72.35)	2.79 (0.99–7.80)
25–29	61,917 (25.62)	9 (19)	14.54 (6.99–26.20)	1.28 (0.42–3.86)
30–34	75,936 (31.42)	10 (21)	13.17 (6.60–23.10)	1.49 (0.51–4.37)
>35	56,544 (23.4)	5 (10)	8.84 (3.17–19.01)	Referent
Total	241,653 (100.00)	48 (100.00)	19.86	NA
Socioeconomic deprivation				
Quintile 5	78,822 (32.6)	26 (54.2)	32.99 (21.88–47.34)	1.89 (0.83–4.30)
Quintile 4	40,440 (16.7)	12 (25)	29.67 (15.89–49.76)	2.52 (1.06–6.02)
Quintile <3	122,391 (50.7)	10 (20.8)	8.17 (4.09–14.33)	Referent
Total	241,653 (100.00)	48 (100.00)	19.86	NA

*Maternal age data were missing for 2 pregnancies, and 2013 area-level New Zealand deprivation index (NZDep2013) socioeconomic deprivation data were missing for 2 additional pregnancies. Therefore, crude incidence rates and relative risks from a multivariable Poisson regression model with ethnicity, age and NZDep2013 quintile in the model were calculated from the 48 cases where complete demographic data were available. The other ethnicity category comprised 3 Asian women and 1 Middle Eastern woman. p<0.0035 for ethnicity, p<0.1115 for age, and p<0.1015 for socioeconomic deprivation. NZDep2013 quintile 5 is the most deprived socioeconomic area, and quintile 1 is the least deprived socioeconomic area. NA, not applicable.

## Discussion

*H. influenzae* has recently been recognized as a rare but major cause of pregnancy-associated invasive disease. In this retrospective study in the Auckland region, accounting for more than one third of the New Zealand population, the overall incidence of pregnancy-associated invasive *H. influenzae* disease was 19.9 cases/100,000 births. Our findings build on those from England and Wales, where the incidence of invasive NTHi infection in pregnant women was 17-fold higher than in nonpregnant women and was strongly associated with preterm birth and a high case-fatality rate (*7*,*9*). However, the mechanism of adverse pregnancy outcomes was unclear; chorioamnionitis was noted in only 7.3% of cases of early-onset neonatal NTHi infection (*9*). Our data indicate that IAI is the probable cause of preterm birth and fetal loss; we found clinical or microbiologic evidence of IAI in 66.7% of our cases. IAI caused by *H. influenzae* has been noted in case reports/series previously (*10**,**11*), but not in a study of this size.

Our data confirm that outcomes of pregnancy-associated invasive *H. influenzae* disease for the fetus or neonate are poor. Adverse pregnancy outcomes (fetal loss, preterm birth, or birth of an infant requiring care in NICU/SCBU) occurred in 94% of pregnancies for which outcome data were available. Only 6% of pregnancies resulted in live birth of an infant not requiring NICU/SCBU care. In contrast, outcomes of pregnancy-associated invasive *H. influenzae* disease for the pregnant woman are generally good. There were no deaths among the 50 women for which data was available, suggesting that this condition can be readily treated by delivery of the fetus and placental tissues, plus administration of antimicrobial drugs.

We found that pregnancy-associated invasive *H. influenzae* infection disproportionately affected Māori persons, who experience a higher burden of many infectious diseases in New Zealand (*17*). Pacific women also had a higher incidence of disease than women of European or other ethnicities, but this difference did not reach statistical significance by multivariable analysis. Potentially relevant to our study are the high rates of sexually transmitted infections in young Māori and Pacific women (*18*). Large ethnic disparities in the incidence of common sexually transmitted infections in New Zealand have persisted, relatively unchanged, in recent years (*19*). In the light of increasing evidence that *H. influenzae* may cause nongonococcal urethritis in men (*20*), one possible hypothesis is that sexually acquired vagino‒cervical *H. influenzae* infection was the immediate precursor of IAI in the women we studied. A high incidence of sexually transmitted infections (*21*) and a high incidence of pregnancy-associated invasive *H. influenzae* disease (*22*) has also been observed in indigenous women in Australia, supporting this proposed mode of infection.

Our study supports IAI as the mechanism by which *H. influenzae* mediates poor pregnancy outcomes. Our findings suggest that IAI is responsible for the major manifestations of pregnancy-associated invasive *H. influenzae* disease. *H. influenzae* is rarely isolated from the genital tracts of pregnant women, having been found in <0.5% of samples from healthy pregnant women (*23**–**25*). We presume that *H. influenzae* infection of the lower genital tract of pregnant women, perhaps acquired as a sexually transmitted infection, or by some other mode of acquisition, places pregnant women at risk for ascending infection; placental infection would then be the route of infection for the fetus. Alterations in hormonal, metabolic, and immune regulation that occur during pregnancy to enable healthy fetal development might result in spread of *H. influenzae* infection from the vagina to the uterine cavity and increase the risk for placental infection (*26*).

Strengths of this study include the analysis of 10 years of data from 4 hospitals and 3 microbiology laboratories caring for demographically diverse populations, with linked outcomes of neonatal and maternal cases. Weaknesses include the retrospective study design, heterogeneity in the quality of the clinical documentation, and missing data. The true burden of pregnancy-associated *H. influenzae* disease, both in our study and in clinical practice, might be underestimated, given that this diagnosis relies on appropriate collection and testing of microbiologic samples. We applied a strict definition of microbiologically confirmed invasive *H. influenzae* disease. It is likely that some women who have signs and symptoms consistent with pregnancy-associated invasive *H. influenzae* disease during the study period did not have adequate microbiologic sampling to enable this diagnosis to be made. Maternal bacteremia was commonly accompanied by concurrent isolation of *H. influenzae* from genital tract or placental specimens, indicating that clinicians were suspicious of IAI as the cause for bacteremia in these women. In contrast, isolation of *H. influenzae* from maternal specimens was uncommon in cases of neonatal bacteremia, perhaps suggesting that the potential for IAI had not been recognized at the time of delivery, resulting in failure to collect appropriate specimens.

Future work should further examine the epidemiology of pregnancy-associated invasive *H. influenzae* disease, assessing whether incidence varies in specific populations, including other indigenous or socioeconomically deprived populations. Rates of genital tract colonization with *H. influenzae* should be quantified in high-risk populations, particularly Māori and Pacific women in New Zealand and indigenous women in Australia. Larger prospective studies should seek to identify factors that predispose to pregnancy-associated invasive *H. influenzae* disease. This approach might identify associations with other sociodemographic variables that our study lacked power to detect. Invasive *H. influenzae* disease should be also considered for pregnant women with signs of chorioamnionitis. Empiric antimicrobial drug treatment in this setting should be with an agent active against *H. influenzae* and other major maternal/perinatal pathogens. *H. influenzae* bacteremia in pregnancy should prompt clinicians to consider intraamniotic infection.

In our study, the overall incidence of pregnancy-associated *H. influenzae* invasive disease was 19.9 cases/100,000 births, similar to the national rate of early-onset neonatal group B *Streptococcus* sepsis in New Zealand (23 cases/100,000 live births) during 2011–2013 (*27*) and higher than the national rate of pregnancy-associated listeriosis in New Zealand (12.3 cases/100,000 live births) during 1997–2016 (*28*). The rates of early-onset group B *Streptococcus* and of pregnancy-associated listeriosis were not higher in those of Māori descent than in persons of European descent (*27*,*28*).

In conclusion, the rates of adverse outcomes in pregnancy-associated invasive *H. influenzae* disease we found were comparable with those for pregnancy-associated listeriosis; fetal loss occurred in 35.4% of cases in our study and in 34% of pregnancy-associated listeriosis cases in New Zealand (*28*). Comparisons across studies using different methods require caution. Nonetheless, our data indicate that, in New Zealand, the burden of *H. influenzae* in pregnancy might be comparable to, or higher than, that seen for pregnancy-associated listeriosis. In addition, the risk for this condition is particularly high for persons of Māori ethnicity.
